# GluN2A Subunit-Containing NMDA Receptors Are the Preferential Neuronal Targets of Homocysteine

**DOI:** 10.3389/fncel.2016.00246

**Published:** 2016-11-01

**Authors:** Dmitry A. Sibarov, Polina A. Abushik, Rashid Giniatullin, Sergei M. Antonov

**Affiliations:** ^1^Laboratory of Comparative Neurophysiology, Sechenov Institute of Evolutionary Physiology and Biochemistry of the Russian Academy of SciencesSaint-Petersburg, Russia; ^2^Department of Neurobiology, University of Eastern FinlandKuopio, Finland; ^3^Laboratory of Neurobiology, Kazan Federal UniversityKazan, Russia

**Keywords:** homocysteine, glutamate, NMDA receptor, desensitization, neurons, primary culture, GluN2A, GluN2B

## Abstract

Homocysteine (HCY) is an endogenous redox active amino acid, best known as contributor to various neurodegenerative disorders. Although it is known that HCY can activate NMDA receptors (NMDARs), the mechanisms of its action on receptors composed of different NMDA receptor subunits remains almost unknown. In this study, using imaging and patch clamp technique in cultured cortical neurons and heterologous expression in HEK293T cells we tested the agonist activity of HCY on NMDARs composed of GluN1 and GluN2A subunits (GluN1/2A receptors) and GluN1 and GluN2B subunits (GluN1/2B receptors). We demonstrate that the time courses of Ca^2+^ transients and membrane currents activated by HCY and NMDA in cortical neurons are drastically different. Application of HCY to cortical neurons induced responses, which in contrast to currents induced by NMDA (both in the presence of glycine) considerably decreased to steady state of small amplitude. In contrast to NMDA, HCY-activated currents at steady state were resistant to the selective GluN2B subunit inhibitor ifenprodil. In calcium-free external solution the decrease of NMDA evoked currents was abolished, suggesting the Ca^2+^-dependent NMDAR desensitization. Under these conditions HCY evoked currents still declined almost to the baseline suggesting Ca^2+^-independent desensitization. In HEK293T cells HCY activated NMDARs of GluN1/2A and GluN1/2B subunit compositions with EC_50_s of 9.7 ± 1.8 and 61.8 ± 8.9 μM, respectively. Recombinant GluN1/2A receptors, however, did not desensitize by HCY, whereas GluN1/2B receptors were almost fully desensitized by HCY. Thus, HCY is a high affinity agonist of NMDARs preferring the GluN1/2A subunit composition. Our data suggest that HCY induced native NMDAR currents in neurons are mainly mediated by the “synaptic type” GluN1/2A NMDARs. This implies that in hyperhomocysteinemia, a disorder with enlarged level of HCY in plasma, HCY may persistently contribute to post-synaptic responses mediated by GluN2A-containing NMDA receptors. On the other hand, HCY toxicity may be limited by desensitization typical for HCY-induced activation of GluN2B-containing extrasynaptic receptors. Our findings, therefore, provide an evidence for the physiological relevance of endogenous HCY, which may represent an effective endogenous modulator of the central excitatory neurotransmission.

## Introduction

L-Homocysteine [2-amino-4-sulfanylbutanoic acid (HCY)] is an endogenous sulfur-containing amino acid involved in synthesis of methionine and cysteine. The normal HCY level in plasma is generally below 16 μM (Shi et al., 2003). However, a deficit of folic acid and vitamins B or the C677T polymorphism of the 5′-10′-methylenetetrahydrofolate reductase gene (a substitution of cytosine for thymine at position 677, C677T) can cause an elevation of HCY level (a condition known as hyperhomocysteinemia, Kowa et al., 2000). High level of HCY have been proposed to contribute to a variety of cardiovascular and neurodegenerative disorders, such as Alzheimer’s and Parkinson’s disease (Kuhn et al., 2001; Kruman et al., 2002; Sachdev, 2005) as well as amyotrophic lateral sclerosis (Zoccolella et al., 2010). Recent data implicate the C677T polymorphism in the pathogenesis of migraine with aura (Moschiano et al., 2008; Lea et al., 2009; Oterino et al., 2010). We previously showed that the high level of HCY led to the neuronal death through activation of NMDA receptors (NMDARs) and mGluR5 (Abushik et al., 2014).

Several studies reported that HCY (Lipton et al., 1997; Ganapathy et al., 2011; Bolton et al., 2013), as well as its derivative, homocysteic acid, studied earlier (Provini et al., 1991; Kilić et al., 1992) is an agonist operating via the glutamate binding sites of the NMDARs. In addition, HCY at relatively high concentrations (above 1 mM HCY) can compete with glycine for the NMDAR co-agonist binding sites (Lipton et al., 1997). Recently, effects of HCY at relatively high concentrations (up to 1 mM) on NMDA and glutamate activated currents transferred through the channels of recombinant NMDARs composed of the GluN1 and GluN2A, GluN1 and GluN2B or GluN1 and GluN2D subunits were studied (Bolton et al., 2013). It has been demonstrated, that HCY can differently modulate peak amplitude of currents activated by NMDA and glutamate and reduce NMDAR desensitization caused by NMDA and glutamate depending on GluN2 subunit compositions (Bolton et al., 2013). Thus, several groups suggested that neuronal NMDARs may represent an important target for the action of elevated HCY (Kim and Pae, 1996; Lipton et al., 1997; Ganapathy et al., 2011; Poddar and Paul, 2013). Nevertheless, the specific features, associated with the action of HCY on NMDARs composed of the “synaptic type” GluN2A subunit and the “extrasynaptic type” GluN2B subunit are still missing.

An accumulation of HCY in the cerebrospinal fluid mentioned above is associated with severe pathologies and dysfunctions of the human central nervous system. As a wide population of patients suffers from hyperhomocysteinemia, a new knowledge of the precise receptor mechanisms of neuronal HCY effects may potentially improve a therapeutic strategy of these diseases. In addition, a discrepancy in mechanisms, by which HCY activates NMDARs composed of different GluN2 subunits may provide important information for NMDAR physiology. Therefore, in order to clarify subunit-specific properties of HCY action on NMDARs we studied its effects on native NMDARs in rat cortical neurons and recombinant GluN1/2A and GluN1/2B receptors expressed in HEK293T cells.

Here, we report that GluN2A subunit-containing NMDARs represent the preferential targets for HCY and mainly contribute to neuronal pathogenesis during hyperhomocysteinemia through activation of synaptic GluN1/2A receptors.

## Materials and Methods

### Primary Culture of Cortical Neurons

The procedure of culture preparation from rat embryos was previously described (Antonov et al., 1998; Mironova et al., 2007). All procedures using animals were in accordance with recommendations of the Federation for Laboratory Animal Science Associations and approved by the local Institutional Animal Care and Use Committees. Wistar rats (provided by the Sechenov Institute’s Animal Facility) 16 days pregnant (overall 12 animals in this study) were sacrificed by CO_2_ inhalation. Fetuses were removed and their cerebral cortices were isolated, enzymatically dissociated, and used to prepare primary neuronal cultures. Cells were used for experiments after 10–15 days in culture (Mironova et al., 2007; Han and Stevens, 2009). Cells were grown in Neurobasal^TM^ culture medium supplemented with B-27 (Gibco-Invitrogen, UK) on glass coverslips coated with poly-D-lysine.

### HEK293T Cells with Recombinant NMDAR

Human embryonic kidney (HEK) 293T cells were maintained as previously described (Qian et al., 2005). HEK293T cells were plated onto 7 mm glass coverslips pretreated with poly-L-lysine (0.2 mg/ml) in 35 mm culture dishes at 1 × 10^5^ cells per dish. 18–24 h after plating cells were transiently transfected with a pcDNA1 plasmid encoding either rat NMDA receptor subunit GluN2A or GluN2B, and pcDNA3.1 plasmids encoding GluN1 and EGFP using FuGene HD reagent (Promega, Madison, WI, USA). Briefly, transfection was performed by adding to each dish 50 μl serum-free medium containing 1 μg total DNA and 2 μl FuGene. The ratio of cDNA used was 1 EGFP: 1 GluN1: 3 GluN2 (A or B). After incubation of cells for 6–8 h the transfection solution was replaced with fresh culture medium containing 200 μM DL-2-amino-5-amino-5-phosphono-valeric acid (DL-AP-5) and 2 mM Mg^2+^ to prevent NMDA receptor mediated-excitotoxicity. Experiments were performed 24–72 h after transfection. Mammalian expression vectors were supplied by Dr. J. W. Johnson (University of Pittsburgh, Pittsburgh, PA, USA).

### Patch Clamp Recordings

Whole-cell patch clamp recordings of membrane currents were performed on cultured rat cortical neurons (10–15 DIV) and HEK293T cells expressing recombinant GluN1/2A or GluN1/2B receptors. We used a MultiClamp 700B patch-clamp amplifier with Digidata 1440A acquisition system controlled by pClamp v10.2 software (Molecular Devices, Sunnyvale, CA, USA). The signal was 8-order low-pass Butterworth filtered at 200 Hz to remove high frequency noise. Acquisition rate was 20000 s^-1^. Micropipette positioning was made with an MP-85 micromanipulator (Sutter Instrument, Novato, CA, USA) under visual control using a Nikon Diaphot TMD microscope (Nikon, Japan). For fast medium exchange we used a BPS-4 fast perfusion system (ALA Scientific Instruments, Farmingdale, NY, USA). The tip of the multichannel manifold was placed at a distance of 0.2 mm from the patched cell, allowing solution exchange in 80 ms. Unless otherwise specified the following extracellular medium was used for recording (external bathing solution, in mM): 140 NaCl; 2.8 KCl; 1.0 CaCl_2_; 10 HEPES, at pH 7.2–7.4. Patch-pipette solution had the following composition (in mM): 120 CsF, 10 CsCl, 10 EGTA, and 10 HEPES. The pH was adjusted to 7.4 with CsOH. Measured osmolarities of the external bathing solution and the patch-pipette solution were 310 and 300 mOsm, respectively. Patch pipettes (2–4 MΩ) were pulled from 1.5-mm (outer diameter) borosilicate standard wall capillaries with inner filament (Sutter Instrument, Novato, CA, USA). Experiments were performed at room temperature (23–25°C). In whole-cell configuration the series resistances did not exceed 10 MΩ. After correction for the liquid junction potential between the Na^+^-containing external bathing solution and the Cs^+^-containing pipette solution of -15 mV the default membrane voltage (V_m_) was set to -55 mV for neurons and -35 mV for HEK293T cells. Functional activity of NMDARs requires binding of both glutamate and a co-agonist, glycine. Unless otherwise stated, to activate NMDARs we applied 50 μM L-HCY (HCY) or 30 μM NMDA with 30 μM L-glycine (Gly).

### Loading of Fluo-3 AM and Ca^2+^ Imaging

Cells were loaded with Fluo-3 AM (4 μM, Life Technologies, Foster City, CA, USA) using conventional protocols as recommended by the manufacturer. In brief, neuronal cultures were incubated with Fluo-3 AM for 45 min in the dark at room temperature. Then, Fluo-3 AM was washed out, and cells were incubated in the external solution for another 30 min in the dark. Coverslips with Fluo-3-loaded cultures were placed in the perfusion chamber, which was mounted on the stage of a Leica TCS SP5 MP inverted microscope (Leica Microsystems, Germany). Fluorescence was activated with 488 nm laser light and emission was measured within the wavelength range from 500 to 560 nm. Images were captured every minute during 60 min experiments. In Ca^2+^ imaging experiments to activated NMDARs 100 μM DL-HCY and 30 μM Gly were used.

### Drugs

Compounds were from Sigma-Aldrich, St. Louis, MO, USA.

### Data Analysis

Quantitative data are expressed as mean ± SEM. ANOVA and Bonferroni multiple comparison methods as well as Student’s two-tailed *t*-test were used for statistical analysis. Number of experiments is indicated by *n* throughout. The data were considered as significantly different based on a confidence level of 0.05. Current measurements were plotted using ClampFit 10.2 (Molecular Devices). The EC_50_ (half-maximal effective concentration for HCY as an agonist) and Hill coefficient (*h*) were estimated by fitting of concentration-response curves with the Hill equation, I_[HCY]_/I_max_ = 1/(1 + EC_50_*^h^*/[HCY]*^h^*), where the I_max_ is the current of maximal amplitude elicited by HCY and I_[HCY]_ are the current amplitudes measured at each [HCY].

## Results

### Amplitude and Dynamic Difference of Neuronal Responses to HCY and NMDA

**FIGURE 1:**
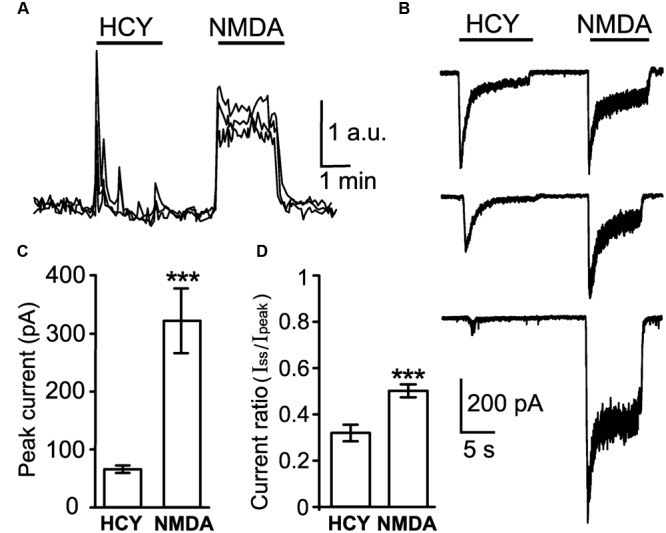
**Comparison of neuronal responses to NMDAR agonists. (A)** Ca^2+^ responses induced by 100 **μ**M DL-HCY and 30 μM NMDA in the presence of 30 μM Gly applied for 2 min to neurons loaded with Fluo-3 AM. Traces represent an overlay of neuronal responses (four neurons). Application protocol is shown above the traces. **(B)** Whole-cell currents activated in neurons by application of 50 μM HCY and 30 μM NMDA in the presence of 30 μM Gly recorded at V_m_ = -55 mV. **(C)** Quantitative comparison of peak amplitudes of currents activated by 50 μM HCY and 30 μM NMDA in the presence of 30 μM Gly recorded from the same neurons (*n* = 57) at V_m_ = -55 mV during paired agonist applications. Data differ significantly (^∗∗∗^*p* < 0.0001, two-tailed Student’s *t*-test). **(D)** Quantitative comparison of steady-state amplitude (I_ss_) to peak amplitude (I_peak_) ratios of currents activated by HCY and NMDA during paired applications. Data differ significantly (^∗∗∗^*p* < 0.0001, two-tailed Student’s *t*-test).

As the starting point for evaluation of agonist properties of HCY, we compared intracellular Ca^2+^ responses ([Ca^2+^]_i_) elicited by NMDA and HCY in non-patched cortical neurons. **Figure 1A** shows that application of 100 μM DL-HCY induced fast Ca^2+^ responses, which quickly decayed to the baseline and often were followed by small repetitive waves. In contrast, application of 30 μM NMDA activated typical sustained [Ca^2+^]_i_ responses of comparable amplitude, that lasted as long as NMDA was present (**Figure 1A**). As the [Ca^2+^]_i_ increase could be determined by Ca^2+^ entry through the NMDARs, voltage gated calcium channels, and/or Ca^2+^ release from intracellular stores, we next explored the action of HCY using patch clamp technique. Consistent with the different shape of Ca^2+^ responses, membrane currents activated by 50 μM HCY differed from currents activated by 30 μM NMDA, both in amplitude and time course. Although in some cells, HCY and NMDA activated currents of comparable peak amplitude (**Figure 1B**, top), the average peak amplitude of currents activated by HCY was significantly less than of currents activated by NMDA (**Figure 1C**). However, the most notable difference was the relative level of the steady-state currents. **Figure 1D** demonstrates that the ratio of steady-state amplitudes to peak amplitudes was significantly smaller for HCY activated currents than for NMDA activated currents. In almost all cases the steady-state current amplitude was much lower when activated by HCY than when activated by NMDA (**Figures 1B,C**). Thus, both Ca^2+^-imaging and patch clamp recording data uncovered essential differences between the actions of HCY and NMDA on native NMDARs in cortical neurons.

### Pharmacological Features of Neuronal Responses to HCY

**FIGURE 2:**
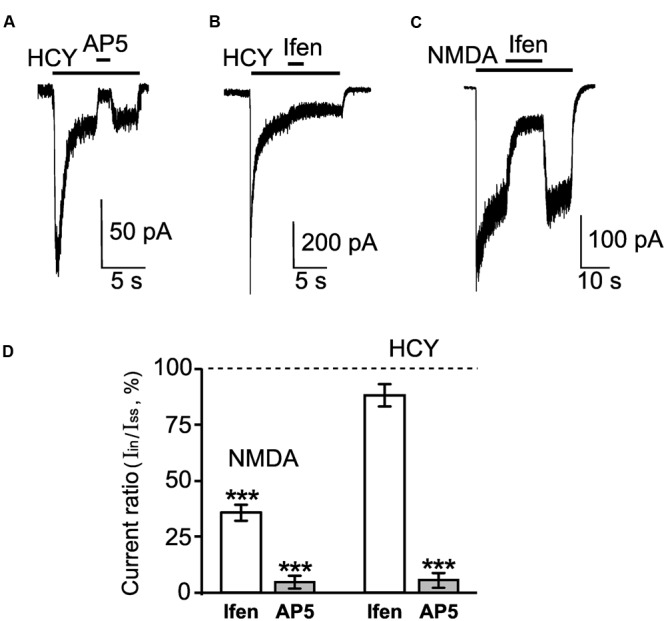
**Pharmacological properties of neuronal currents activated by HCY and NMDA. (A)** Representative current trace recorded in a neuron at V_m_ = -55 mV activated by 50 μM HCY + 30 μM Gly. After current reached steady state, 100 μM AP-5 was applied. The application protocol is shown above the trace. **(B)** Representative current trace recorded in a neuron at V_m_ = -55 mV activated by 50 μM HCY + 30 μM Gly. After current reached steady state, 5 μM ifenprodil (ifen) was applied. The application protocol is shown above the trace. **(C)** Representative current trace recorded in a neuron at V_m_ = -55 mV activated by 30 μM NMDA + 30 μM Gly. After current reached steady state, 5 μM ifenprodil (ifen) was applied. The application protocol is shown above the trace. **(D)** Quantitative comparison of inhibition of NMDA-activated currents caused by 5 μM ifenprodil (ifen, *n* = 18) and 100 μM AP-5 (*n* = 24) and inhibition of HCY-activated currents caused by 5 μM ifenprodil (ifen, *n* = 26) and 100 μM AP-5 (*n* = 9). Ordinate axis – ratio of the current amplitude obtained during inhibition (I_in_) to the steady-state current amplitude (I_ss_) obtained without antagonists. Data differ significantly from steady-state current control amplitudes (^∗∗∗^*p* < 0.0006, ANOVA, *post hoc* Bonferroni test).

We further compared pharmacological characteristics of currents activated by HCY and NMDA. An antagonist of mGluR5, 3-((2-methyl-4-thiazolyl)ethynyl)pyridine (MTEP), did not affect HCY-induced currents under experimental conditions used here, suggesting that activation of mGluR5 does not contribute to the currents activated by HCY (data not shown). Competitive antagonist of NMDARs (2R)-amino-5-phosphonopentanoate (AP-5), caused almost complete block of HCY-induced currents (**Figures 2A,D**) suggesting that the currents were indeed transferred through the NMDAR channels. Notably, the currents activated by HCY at steady state were resistant to the action of ifenprodil (**Figures 2B,D**). In contrast, this GluN2B subunit selective inhibitor (Williams, 1993) efficiently blocked currents activated by NMDA (**Figures 2C,D**).

Taken together, these experiments demonstrated considerable pharmacological differences between NMDA and HCY as agonists of NMDARs.

### NMDAR Desensitization Induced by NMDA and HCY in Neurons

Since different agonists of NMDARs can induce NMDAR desensitization of different onset and offset rates (Lester and Jahr, 1992), we further studied desensitization of NMDARs caused by NMDA and HCY in cortical neurons.

**FIGURE 3:**
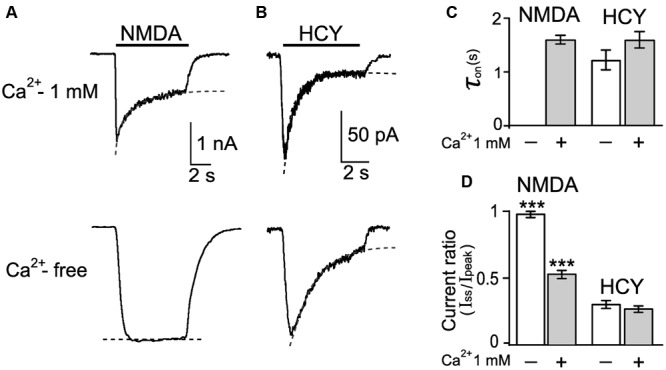
**Desensitization of native NMDARs caused by NMDA and HCY. (A)** Currents activated by 30 μM NMDA + 30 μM Gly in neurons recorded at V_m_ = -55 mV in the presence and absence of Ca^2+^ in the external bathing solutions. Dotted lines represent fits of a single exponential function to current decays. **(B)** Currents activated by 50 μM HCY + 30 μM Gly in neurons recorded at V_m_ = -55 mV in the presence and absence of Ca^2+^ in the external bathing solutions. Dotted lines represent fits of single exponential function to current decays. **(C)** Quantitative comparison of NMDAR desensitization onsets evoked by NMDA and HCY in the presence and absence of Ca^2+^ in the external bathing solution. τ_on_ was measured as the time constant of current decay to the steady-state level. Data do not differ (*p* > 0.36, ANOVA, *n* = 10). **(D)** Quantitative comparison of NMDAR desensitization evoked by NMDA and HCY in the presence and absence of Ca^2+^ in the external bathing solution. Ordinate axis – ratio of amplitudes obtained at steady-state (I_ss_) and peak (I_peak_) current as a measure of desensitization. Values differ significantly from data, obtained with HCY (^∗∗∗^*p* < 0.001, ANOVA, *post hoc* Bonferroni test, *n* varied from 16 to 56 for different data sets).

Currents activated by NMDA declined to a steady-state level because of receptor desensitization (**Figure 3A**). The time constant of desensitization onset (τ_on_) was measured by fitting the decay of NMDAR mediated currents to steady state with a single exponential function (**Figure 3A**). In the presence of Ca^2+^ in the external bathing solution the τ_on_ value for NMDA induced currents was 1.6 ± 0.1 s (*n* = 36).

In the case of HCY, currents also declined to the steady-state level (**Figure 3B**). The τ_on_ for HCY was 1.6 ± 0.2 s (*n* = 47) and did not differ significantly from NMDAR desensitization caused by NMDA (*p* > 0.8, Student’s two-tailed *t*-test, **Figure 3C**).

In Ca^2+^-free external bathing solution, however, the currents activated by NMDA did not decrease in time, revealing the lack of desensitization (**Figures 3A,D**). In sharp contrast, the onset of desensitization caused by HCY remained unchanged (τ_on_ = 1.2 ± 0.2 s, *n* = 34, **Figures 3B–D**). These data demonstrated that NMDA-induced responses were characterized by Ca^2+^-dependent desensitization (Legendre et al., 1993; Zhang et al., 1998; Sibarov et al., 2015), whereas desensitization of HCY-induced responses was almost Ca^2+^-independent.

**FIGURE 4:**
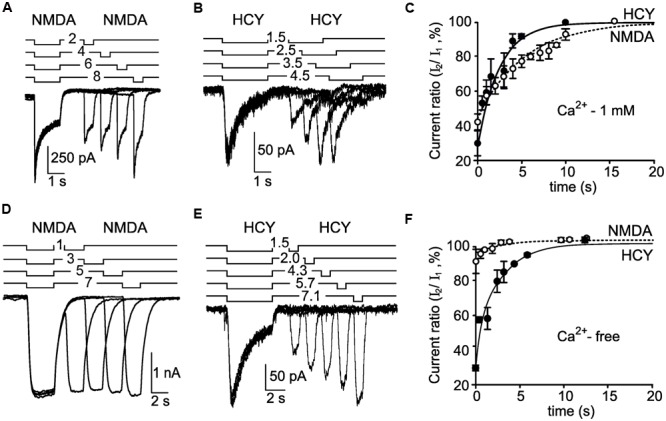
**Recovery from desensitization induced by NMDA and HCY. (A)** An overlay of currents activated by 30 μM NMDA + 30 μM Gly using the paired application protocol (shown above the traces) recorded in the neuron at V_m_ = -55 mV in the presence of Ca^2+^ in the external bathing solution. **(B)** An overlay of currents activated by 50 μM HCY + 30 μM Gly using the paired application protocol (shown above the traces) recorded in the neuron at V_m_ = -55 mV in the presence of Ca^2+^ in the external bathing solution. **(C)** The dependence of the peak current amplitude achieved during the second agonist application on the duration of delay between applications. Ordinate axis – ratio of the peak amplitudes of the current elicited by the second application (I_2_) to the current elicited by the first application (I_1_). The peak amplitude of currents activated by the first application was taken for 100%. Open circles – the data obtained by NMDA applications (*n* = 6 for each point). Filled circles – the data obtained by HCY applications (*n* = 5 for each point). Lines represent fits of a single exponential function to the data points. **(D)** An overlay of currents activated by 30 μM NMDA + 30 μM Gly using the paired application protocol (shown above the traces) recorded in the neuron at V_m_ = -55 mV in Ca^2+^-free media. **(E)** An overlay of currents activated by 50 μM HCY + 30 μM Gly using the paired application protocol (shown above the traces) recorded in the neuron at V_m_ = -55 mV in Ca^2+^-free media. **(F)** The dependence of the peak current amplitude achieved during the second agonist application on the duration of delay between applications. Ordinate axis – ratio of the peak amplitudes of the current elicited by the second application (I_2_) to the current elicited by the first application (I_1_). The peak amplitude of currents activated by the first application was taken for 100%. Open circles – the data obtained by NMDA applications (*n* = 6 for each point). Filled circles – the data obtained by HCY applications (*n* = 4 for each point). Lines represent fits of a single exponential function to the data points.

In order to estimate the time constant of recovery from desensitization induced by NMDA and HCY for native NMDARs we used a double application protocol consisting of 4–12 repeats. In this protocol, the second application of the agonists in the next repeat was applied with an incrementing delay of 1–2 s from the first. The increment duration was chosen to achieve a best resolution of the desensitization offset (**Figures 4A,B**). The recovery from desensitization was measured as an increase of current amplitudes in the sequence of second agonist applications which was well-fitted by a single exponential function with a time constant (τ_off_) of the desensitization offset (**Figure 4C**). Whereas, the τ_on_ values of the native NMDAR desensitization onset caused by NMDA and HCY were similar (**Figure 3C**), the τ_off_ value for NMDA elicited currents was significantly larger, than those for HCY (*p* < 0.0001, Student’s two-tailed *t*-test) and were 6.3 ± 1.1 (*n* = 6) and 2.5 ± 0.5 s (*n* = 5), respectively (**Figure 4C**).

The removal of Ca^2+^ from the external bathing solution nearly abolished the amplitude decrease of NMDA-activated currents (**Figure 4D**) demonstrating the Ca^2+^ dependence of NMDAR desensitization. In contrast the decrease of currents activated by HCY remained unchanged (**Figure 4E**) in the Ca^2+^-free media suggesting that native NMDAR desensitization evoked by HCY is not Ca^2+^- dependent. Moreover, the τ_off_ value of HCY induced desensitization (τ_off_ = 2.7 ± 0.7 s, *n* = 4, **Figure 4F**) obtained in the Ca^2+^-free media was similar to the τ_off_ value found in the presence of Ca^2+^ in the external bathing solution (τ_off_ = 2.5 ± 0.5 s, *n* = 5, **Figure 4C**) and did not differ significantly (*p* > 0.85, Student’s two-tailed *t*-test).

Overall, the above experiments reveal substantial kinetic, amplitude, and pharmacological differences between currents activated by NMDA and HCY in cortical neurons as well as specific properties of desensitization with respect to Ca^2+^ dependence of this phenomenon.

### HCY Induces Fast Desensitization of Recombinant GluN1/2B NMDARs

Because cortical neurons in primary culture typically express NMDARs of GluN1/2A and GluN1/2B subunit compositions (Zhong et al., 1994; Mizuta et al., 1998) we hypothesized that HCY when activates these receptor subtypes may causes different kinetics of activation and desensitization.

**FIGURE 5:**
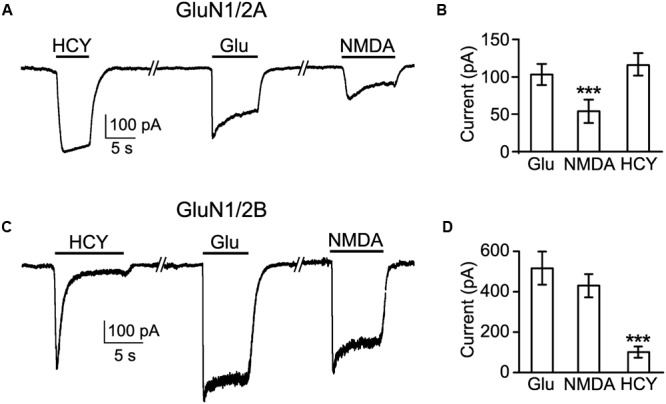
**Comparison of currents activated by HCY in HEK293T cells expressing recombinant GluN1/2A and GluN1/2B receptors. (A)** Currents activated by 50 μM HCY, 100 μM Glu, and 30 μM NMDA in the presence of 30 μM Gly recorded in HEK293T cells at V_m_ = -35 mV expressing NMDARs of GluN1/2A subunit compositions. **(B)** Histogram of the steady-state amplitudes of currents activated by these agonists in the same cells. The data are significantly different (^∗∗∗^*p* < 0.0001, ANOVA, *post hoc* Bonferroni test, *n* = 37). **(C)** Currents activated by 50 μM HCY, 100 μM Glu, and 30 μM NMDA in the presence of 30 μM Gly recorded in HEK293T cells at V_m_ = -35 mV expressing NMDARs of GluN1/2B subunit compositions. **(D)** Histogram of the steady-state amplitudes of currents activated by these agonists in the same cells. The data are significantly different (^∗∗∗^*p* < 0.0001, ANOVA, *post hoc* Bonferroni test, *n* = 57).

To get more clues in the mechanism of HCY activation of NMDARs, we performed experiments on recombinant GluN1/2A and GluN1/2B receptors expressed in HEK293T cells. 50 μM HCY applied to cells expressing diheteromeric GluN1/2A receptors caused robust inward currents, which did not reveal decrease of amplitude during 10 s applications, suggesting a lack of GluN1/2A receptor desensitization (**Figure 5A**). Application of 100 μM Glu (a saturating concentration for GluN1/2A receptor, see Traynelis et al., 2010), induced currents which were similar in amplitude to those activated by HCY (**Figure 5B**). In the case of Glu, these currents largely decreased to steady state because of the receptor desensitization (**Figure 5A**). For GluN1/2A receptors, the application of 30 μM NMDA induced currents that were much smaller than those activated by HCY or Glu. Mean amplitude of NMDA activated currents differed from mean amplitudes of HCY and Glu activated currents significantly (*p* < 0.0001, ANOVA, *post hoc* Bonferroni test, *n* = 37, **Figures 5A,B**), because of the NMDA concentration used in these experiments is much smaller than a saturating one. The data obtained with Glu and NMDA are consistent with known efficacy and EC_50_ of Glu and NMDA as agonists of GluN1/2A NMDARs (for review, see Traynelis et al., 2010).

In contrast to GluN1/2A receptors, applications of 50 μM HCY to cells expressing diheteromeric GluN1/2B receptors caused currents that initially peaked and then declined to steady state of small amplitude (**Figure 5C**). 100 μM Glu and 30 μM NMDA induced large GluN1/2B mediated currents (**Figure 5C**) that did not differ significantly in their steady-state amplitudes (**Figure 5D**). The steady-state current amplitudes achieved during applications of HCY were very small in comparison to currents obtained in the presence of Glu and NMDA and differed from them significantly (*p* < 0.0001, ANOVA, *post hoc* Bonferroni test, *n* = 57). The data obtained with respect to Glu and NMDA are consistent with the known efficacy and EC_50_ of these agonists to binding sites on GluN1/2B NMDARs (for review, see Traynelis et al., 2010).

Thus, HCY is an effective agonist of NMDARs of both GluN1/2A and GluN1/2B subunit compositions. GluN1/2A receptors, however, do not exhibit desensitization during activation by HCY, whereas the activation of GluN1/2B receptors by HCY was accompanied by fast and close to complete desensitization.

### HCY as High Affinity Agonist of Recombinant GluN1/2A NMDARs

**FIGURE 6:**
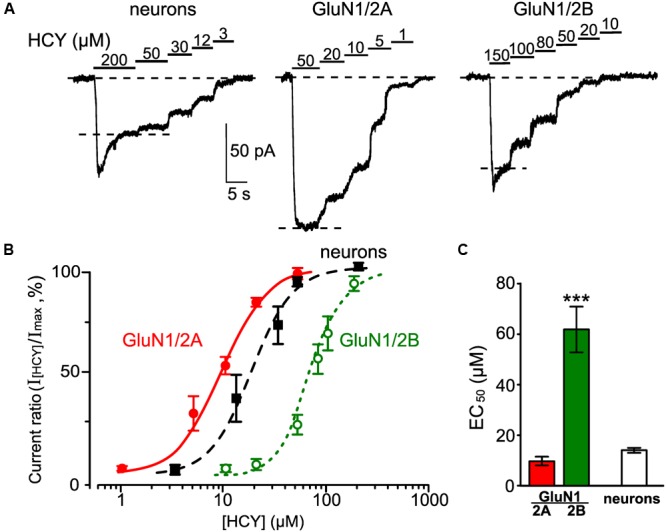
**Measurements of EC_50_ for HCY as an agonist of native NMDARs and recombinant NMDARs of GluN1/2A and GluN1/2B subunit compositions. (A)** Currents elicited by the indicated HCY concentrations in the presence of 30 μM Gly recorded in cortical neurons at V_m_ = -55 mV and in HEK293T cells expressing recombinant GluN1/2A and GluN1/2B NMDARs at V_m_ = -35 mV. Application protocols are indicated above the records. **(B)** Concentration-activation curves for HCY obtained for currents mediated by native NMDARs (filled squares) and recombinant NMDARs of GluN1/2A (filled circles) and GluN1/2B (open circles) subunit compositions. Ordinate axis – ratio of amplitudes obtained at steady state in the presence of different HCY concentrations (I_[HCY]_) to the maximal amplitude (I_max_). Solid lines indicate fits of the data with the Hill equation. **(C)** Quantitative comparison of EC_50_s for HCY activation of native and recombinant GluN2A- and GluN2B-containing NMDARs. Data are significantly different (^∗∗∗^*p* < 0.0001, ANOVA, *post hoc* Bonferroni test, *n* = 6–10).

In further experiments, we measured the potency (EC_50_ values) for activation of HCY currents mediated by native and recombinant GluN1/2A and GluN1/2B NMDARs. First, a saturating concentration of HCY in the presence of 30 μM Gly was applied to neurons or HEK293T cells expressing GluN1/2A and GluN1/2B NMDARs. Then the HCY concentration was decreased until currents could not be activated (**Figure 6A**). For each of NMDAR type the progressive lowering of HCY concentration caused a decrease of NMDAR current amplitude measured at steady state (**Figure 6A**). The dependence of the steady-state current amplitude on HCY concentration is shown in **Figure 6B**. Fits of the Hill equation to the data revealed the apparent dissociation constants (EC_50_) for HCY with NMDARs and the Hill coefficients (*h*) which were 14.4 ± 1.3 μM and *h* = 1.8 ± 0.8 (*n* = 6) for native NMDARs, 9.7 ± 1.8 μM and *h* = 1.6 ± 0.5 (*n* = 10) for GluN1/2A receptors, and 61.8 ± 8.9 μM and *h* = 2.1 ± 0.3 (*n* = 10) for GluN1/2B receptors. Whereas, the HCY EC_50_ values for GluN1/2A and native receptors do not differ significantly, they are significantly smaller than the EC_50_ value for GluN1/2B receptors (*p* < 0.0001, ANOVA, *post hoc* Bonferroni test, **Figure 6C**). Therefore, HCY can be considered a high potency agonist of NMDARs composed by the GluN1/2A subunits. The similarity between the HCY EC_50_ values obtained on the native and GluN1/2A NMDARs, may suggest that the currents, activated by HCY in neurons, at the steady state are predominantly mediated by diheteromeric GluN1/2A NMDARs.

## Discussion

The main finding of this study is that the endogenous amino acid, HCY, implicated in various brain diseases preferentially activates “synaptic type” GluN2A subunit-containing NMDA receptors with specific kinetics properties including limited receptor desensitization.

### HCY Signaling in Brain

Homocysteine that accumulates in plasma during hyperhomo-cysteinemia can potentially lead to the death of cortical neurons (Lipton et al., 1997; Abushik et al., 2014), retinal ganglion cells (Ganapathy et al., 2011) and trigeminal neurons (Abushik et al., 2014). This type of excitotoxicity is likely mediated by activation of native NMDARs (Lipton et al., 1997; Ganapathy et al., 2011; Bolton et al., 2013; Abushik et al., 2014).

The goal of our study was to clarify the mechanism of HCY action on cortical neurons. In order to succeed in addressing this goal, we compared HCY action on recombinant NMDARs of known composition. GluN1/2A and GluN1/2B receptors in different regions of adult brain exhibit preferentially synaptic and extrasynaptic locations, respectively, which are subjected for activity-dependent modulation and may change during pathogenesis (Paoletti et al., 2013). Notably, cortical neurons vary substantially with respect to GluN2A versus GluN2B expression ratio (Tovar and Westbrook, 1999; Traynelis et al., 2010). Our data in general are consistent with the view that HCY is an agonist of NMDAR glutamate binding sites (Lipton et al., 1997; Ganapathy et al., 2011; Bolton et al., 2013). This would suggest that neuronal responses of NMDARs to HCY, Glu or NMDA used at equally potent concentrations should have similar amplitudes and time courses. However, in this study we found that HCY-induced intracellular Ca^2+^ responses as well as NMDAR-mediated currents differ greatly with respect to their amplitudes and time courses from responses to NMDA obtained on the same neurons. In contrast to NMDA, which activated sustained Ca^2+^ intracellular responses, HCY induced fast oscillatory intracellular Ca^2+^ transients which peaked and than briefly declined to the control [Ca^2+^]_i_. Currents activated by HCY also varied greatly by amplitudes between neurons, being generally much smaller (on average about 1/6), than the corresponding NMDA induced currents. Moreover, pharmacological analysis indicated that the steady-state currents, activated by HCY, were resistant to ifenprodil, a GluN2B selective inhibitor of NMDARs (Williams, 1993). Most striking, unlike Ca^2+^-dependent NMDA-induced desensitization, the NMDAR desensitization evoked by HCY did not depend on extracellular Ca^2+^. Thus, Ca^2+^-independent HCY desensitization could be defined as a ligand-dependent NMDAR desensitization (Lester and Jahr, 1992).

Taken together, these observations suggest that neuronal NMDAR currents, transferred through channels of GluN1/2A and GluN1/2B receptors, have very different kinetics and pharmacological sensitivity when activated by HCY and NMDA.

### Role of Subunit Composition

Our experiments on recombinant GluN1/2A and GluN1/2B NMDARs expressed in HEK293T cell disclosed the peculiarities of complex desensitization kinetics when native NMDARs were activated by HCY and NMDA. We found that HCY is a high potency agonist (EC_50_ is about 9 μM) of GluN1/2A receptors. Remarkably, during HCY activation these receptors do not undergo desensitization. In contrast to GluN1/2A receptors, HCY is a low potency agonist of GluN1/2B receptors (EC_50_ is about 70 μM). Furthermore, HCY promotes fast desensitization of GluN2B-containing receptors providing a low-level steady-state current. Recently, effects of HCY on currents activated by NMDA and Glu mediated by different recombinant NMDARs (containing GluN2A or GluN2B or GluN2C) were studied (Bolton et al., 2013). In agreement with our study the HCY effects appeared to be dependent on GluN2 subunits, suggesting deviations of the HCY effects on different GluN2. Indirectly this observation supports conclusions drown here.

**FIGURE 7:**
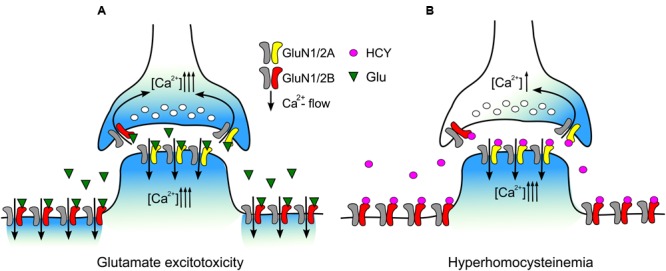
**Schematics of the data interpretation. (A)** During excitotoxic episodes Glu activates both extrasynaptic GluN2B-containng NMDARs and synaptic GluN2A-containing NMDARs. Entry of external Ca^2+^ into neurons through open NMDAR channels causes intracellular Ca^2+^ overload. **(B)** During hyperhomocysteinemia HCY desensitizes extrasynaptic GluN2B-containng NMDARs limiting intracellular Ca^2+^ accumulation in the extrasynaptic regions. Synaptic GluN2A-containing NMDARs are kept activated by HCY that allows intracellular Ca^2+^ accumulation in post-synaptic regions and presynaptic terminal inducing synaptic Ca^2+^ oscillations.

Our data may allow plausible explanation of peculiarities in HCY action that determine amplitude and shape of native NMDAR currents in cortical neurons (**Figure 7**). Both subtypes of NMDARs contribute to currents activated by NMDA (**Figure 7A**) and, most likely, to the peak current activated by HCY. In case of HCY the peak current declines quickly because of the GluN1/2B receptor desensitization caused by HCY. Therefore, GluN1/2B receptors can contribute little to depolarization and Ca^2+^ transients induced by HCY in neurons during long-lasting periods of action (**Figure 7B**). As a result the steady-state currents activated in neurons by HCY are generally transferred through the channels of GluN1/2A receptors, which do not undergo desensitization. Our observation that HCY activated currents at steady state are resistant to ifenprodil is consistent to this explanation. In addition, because the GluN1/2B versus GluN1/2A expression ratio varies substantially between neurons the amplitudes of peak and steady-state components of currents activated by HCY could demonstrate considerable variability.

### Pathophysiological Implications

The data predict that HCY may have different neuronal targets depending on NMDAR subunit compositions. One major distinction between the two receptor subtypes is that the GluN2A-containing NMDARs are thought to occur in post-synaptic regions and have exclusively synaptic location (Tovar and Westbrook, 1999; Momiyama, 2000; Steigerwald et al., 2000; Dalby and Mody, 2003; de Armentia and Sah, 2003, see also **Figure 7**). In opposite, NMDARs, which are supposed to contain the GluN2B subunit, are distributed over large extrasynaptic somatic and dendritic areas and mediate the synaptic cross talk sensing the neurotransmitter spillover during excessive synaptic activity (Scimemi et al., 2004). Interestingly, GluN2A- and GluN2B-containing NMDARs have been linked to different intracellular cascades contributing in LTP and LTD and also leading to preferential triggering neuroprotection and neurodegeneration, respectively (Hardingham and Bading, 2010; Gladding and Raymond, 2011; Stark and Bazan, 2011). Given the preferential contribution of GluN2A-containing NMDARs to the HCY effects, this amino acid is expected to be much less efficient neurotoxicant, than NMDA. In consistence to achieve the similar levels of apoptosis in primary culture of rat cortical neurons a 24-h exposure to HCY was required (Abushik et al., 2014), instead of a 4-h exposure to NMDA (Sibarov et al., 2012). Unlike NMDA-induced excitotoxicity is sensitive to NMDAR inhibitors (Church et al., 1988; Mironova et al., 2007), apoptosis caused by HCY is highly sensitive to the mGluR5 antagonist MTEP (Abushik et al., 2014). The latter suggests that mGluR5 receptors are most important players in triggering HCY excitotoxicity, rather than NMDARs. Based on current observations, we suggest that HCY preferentially increases the synaptic excitability activating the GluN2A-containing NMDARs as well as mGluR5 which can functionally interact with NMDARs at the pre- and post-synaptic compartments (Sylantyev et al., 2013). In contrast to the GluN2A-containing NMDARs, the effects mediated by the GluN2B-containing receptors are expected to be inhibited in the presence of HCY due to essential desensitization (**Figure 7**). This may have some functional consequences interfering with the development of LTD and affecting the neurotransmitter spillover (Scimemi et al., 2004).

The range of HCY concentrations that activates the GluN2A-containing receptors (EC_50_ about 9 μM) corresponds to the normal level of endogenous HCY, which varies from 5 to 15 μM in human plasma (Shi et al., 2003). Notably, during hyperhomocysteinemia the concentration of this amino acid can rise up to 200 μM (Fritzer-Szekeres et al., 1998) making it likely that HCY can contribute to persistent (due to low desensitization) activation of synaptic type NMDARs. Based on our data, the neurotoxic profiles of NMDA or glutamate (**Figure 7A**) and HCY (**Figure 7B**) should be considerably different. In excitotoxicity glutamate activates GluN1/2A and GluN1/2B with similar kinetics and can cause Ca^2+^ overload of extrasynaptic as well as synaptic regions of neurons (**Figure 7A**). In contrast, in hyperhomocysteinemia HCY activates synaptic GluN1/2A receptors which may cause Ca^2+^ accumulation in spines and presynaptic terminals (**Figure 7B**). In addition HCY desensitizes mainly extrasynaptic GluN1/2B receptors, thus limiting Ca^2+^ accumulation in the extrasynaptic regions (**Figure 7B**). Our findings, therefore, provide molecular mechanisms of HCY neuronal effects and a corroborating evidence for the physiological relevance of endogenous HCY, which may allow understanding the role of this amino acid in modulation of the excitatory neurotransmission and neurotoxicity.

## Author Contributions

Experimental work and data acquisition (DS, PA); data analysis and preparation of figures (DS, PA, SA); study design/interpretation and drafting of manuscript (DS, RG, SA); final approval of manuscript (DS, PA, RG, SA).

## Conflict of Interest Statement

The authors declare that the research was conducted in the absence of any commercial or financial relationships that could be construed as a potential conflict of interest.
